# Abnormal Expression of Fgf9 during the Development of the Anorectum in Rat Embryos with Anorectal Malformations

**DOI:** 10.1155/2019/1986196

**Published:** 2019-07-11

**Authors:** Huiying Liu, Hailan Zhang, Meng Li, Hongzhong Tian, Xiaobing Tang, Yuzuo Bai

**Affiliations:** Department of Pediatric Surgery, Shengjing Hospital, China Medical University, Shenyang, China

## Abstract

The study objective was to investigate the role of fibroblast growth factor 9 (Fgf9) in normal and anorectal malformation (ARM) embryos during the development of the anorectum. Fgf9 expression was assayed in both normal rat embryos and embryos with ARM induced by exposure to ethylenethiourea (ETU) on embryonic day 10 (E10). Fgf9 expression was assayed by immunohistochemical staining, Western blotting, and real-time quantitative polymerase chain reaction (qRT-PCR). Immunohistochemical staining revealed spatiotemporal changes in Fgf9 expression between E13 and E16. Fgf9-positive cells predominated in the mesenchyme of the cloaca on E13 and E14 and at the fusion site of the urorectal septum and cloacal membrane, rectal epithelium, and anal membrane on E15. Fgf9-positive cells were obviously decreased after the anal membrane ruptured on E16. Fgf9-positive staining was significantly decreased in embryos with ARM compared with normal embryos from E13 to E15. The results of Western blots and qRT-PCR were consistent, with significantly increased Fgf9 expression in the hindgut and rectum of normal embryos than in embryos with ARM from E13 to E15. However, there was no difference between the two groups on E16. These results suggested that the anorectal embryogenesis might depend on the induction of Fgf9 signal. The expression of Fgf9 was downregulated in ETU-induced ARM embryos, which might be related to the development of ARM.

## 1. Introduction

Anorectal malformations (ARM) are congenital malformations that occur in approximately 1 in 2500–3500 live births worldwide [1, 2] and are usually associated with multiple malformations. The clinical presentations vary from anal stenosis to anorectal fistula to persistent cloaca [3]. Although newborn care and surgical techniques progress, many ARM patients still need to accept challenges for a long time including the bowel and bladder dysfunction, sexual dysfunction, and social psychological problems [4, 5]. The etiology, embryology, and pathogenesis of ARM are poorly understood and also controversial. Current evidences show that ARM is a complex polygenic disease and links the Hox gene family, the sonic hedgehog signaling, the Wnt signaling pathway, bone morphogenetic protein 4 (Bmp4), and fibroblast growth factor 10 (Fgf10) [6–10].

Fgfs comprise a 22-member gene family encoding heparan sulfate-binding proteins and regulator of cell growth and embryonic development following binding to fibroblast growth factor receptors (Fgfrs). Previous studies have documented the abnormal expression of the Fgf10 and Fgfr2b genes in rat embryos with ARM [10, 11], but the roles of the other members of the Fgf gene family in the formation of ARM have not been studied. There are evidences that Fgf9 and Fgf10 reciprocal epithelial-mesenchymal signals are indispensable during some organogenesis [12, 13]. Fgf9 activity during embryonic development of ARM has not yet been demonstrated. In order to reveal the regulation effect of Fgf9 on ARM, we analyzed the distribution and the expression level of Fgf9 from E13 to E16 in rat embryos with ethylenethiourea- (ETU-) induced ARM by immunohistochemistry, Western blotting, and real-time quantitative polymerase chain reaction (qRT-PCR).

## 2. Materials and Methods

### 2.1. Model Preparation and Specimen Collection

The study was approved by the animal ethics committee of Shengjing Hospital Affiliated with China Medical University. The procedure for creating the ARM fetal rat model has been described previously [14]. Fifty-five time-mated pregnant rats were randomly assigned to either a normal or an ARM group and gavage-fed, either a single 125 mg/kg dose of 1% ETU (Aldrich Chemical Co., Penzberg, Germany) or an equal volume of saline on embryonic day 10 (E10). On E0, sperm was visible in a vaginal smear after overnight mating. Cesarean sections were performed on E13–16, and about one-third of the recovered embryos were fixed in 4% paraformaldehyde for 24–48 hours. The embryos were then dehydrated and embedded in paraffin, and 3.5 *μ*m serial sagittal sections were cut and mounted on slides in preparation for immunohistochemical staining. For Western blot and qRT-PCR assays, the hindgut and rectum of remaining embryos were dissected under magnification, frozen immediately in liquid nitrogen, and stored at −80°C until use.

### 2.2. Immunohistochemical Staining

Immunohistochemical staining was performed as described previously [15] using an S-P high-sensitivity Kit (Maixin Biotechnology, Fuzhou, China). For antigen retrieval, slides were heated in boiling 0.01 mol/L pH 6 citric acid buffer for 10 min. After cooling to room temperature, nonspecific binding sites were blocked by 3% peroxide enzyme inhibitor and normal goat serum. Tissue sections were incubated overnight at 4°C with an anti-Fgf9 rabbit polyclonal antibody at 1 : 150 dil (Abcam, Cambridge, UK). The next morning, the sections were allowed to incubate with biotinylated goat anti-rabbit IgG secondary antibody for 20 min at room temperature. Color was developed with 3,3′-diaminobenzidine (ZsBio, Beijing, China), and the sections were counterstained with hematoxylin. The tissues were observed by light microscopy and photographed with a digital microscope camera (Nikon Eclipse Ci, Tokyo, Japan). Negative controls were prepared either by omitting the primary or secondary antibody or by incubating with the equivalent concentrations of nonimmune rabbit antiserum.

### 2.3. Protein Extraction and Western Blot Assay

Tissue collected from the anus and rectum of the ARM model and normal rat embryos was pooled and sonicated in double-distilled H_2_O containing protease inhibitors. Protein extracts (50 *μ*g) were denatured by heating at 90°C for 5 min and then stored at −80°C refrigerator until used. Protein samples were separated by sodium dodecyl sulfate-polyacrylamide gel electrophoresis (SDS-PAGE; Beyotime, Shanghai, China), transferred to the polyvinylidene fluoride (PVDF) membranes (Millipore, Billerica, MA, USA), blocked with 5% fat-free milk in Tris-buffered saline for 1.5 h at room temperature, and incubated overnight at 4°C with primary anti-Fgf9 rabbit polyclonal antibody (Abcam, Cambridge, UK; 1 : 1000 dil). The membranes were incubated with horseradish peroxidase-conjugated goat anti-rabbit secondary antibody (Beyotime, Shanghai, China; 1 : 1000 dil) for 2 h at room temperature. A chemiluminescent substrate kit (BeyoECL Star; Beyotime, Shanghai, China) was used to detect the immunostained bands. In each lane, *β*-actin was used as an internal standard to normalize protein expression.

### 2.4. Nucleic Acid Extraction, Reverse Transcription, and RT-PCR

A TRIzol (Invitrogen Life Technologies, Carlsbad, CA, USA) reagent was used to extract total RNA from anus and rectum tissues collected from normal and ARM embryos. The purity of the total RNA isolates was determined by the 260 : 280 nm absorbance ratio, which was expected to be between 1.8 and 2. The extracted RNA was stored at −80°C. Reverse transcription was performed with a commercially available kit (Takara, Dalian, China). The Fgf9 primers used for qRT-PCR were forward 5′-GCAGTCACGGACTTGGATCATTTA-3′ and reverse 5′-TCCACACCACG AATGCTGAC-3′. Expression of the *β*-actin housekeeping gene (Takara, Dalian, China) was used as an endogenous control. A LightCycler (Takara, Dalian, China) total reaction system and SYBR Green PCR Master Mix (Takara, Dalian, China) were used. The reaction program assayed 20 *μ*L of the sample and consisted of predenaturation at 95°C for 30 s, 5 s of denaturation at 95°C, 30 s of annealing at 60°C, and 40 cycles. After amplification, the system (Applied Biosystems 7500, Singapore) automatically generated the CT values of each sample. The calculation of 2^−*ΔΔ*CT^ values was used to compare gene expression in normal and ARM tissues.

### 2.5. Statistical Analysis

The GraphPad Prism 6.x.C was used for the statistical analysis. Differences in Fgf9 expression in normal and ARM tissues were compared by a *t*-test. Results were expressed as means ± standard deviation. *P* values < 0.05 were considered statistically significant.

## 3. Results

### 3.1. General Observations

No malformations were observed in the 223 embryos from normal rats. The numbers of embryos at different gestational ages are shown in Table 1. All 271 ETU-treated embryos had a short or absent tail; none of the embryos died in utero. ARM was present in 79% of the ETU-treated embryos, and the most frequent malformations were persistent cloaca or rectourethral fistula under the light microscope. The numbers of embryos in the ARM group at different gestational ages are shown in Table 2.

### 3.2. Immunohistochemical Results

**Figure 1:**
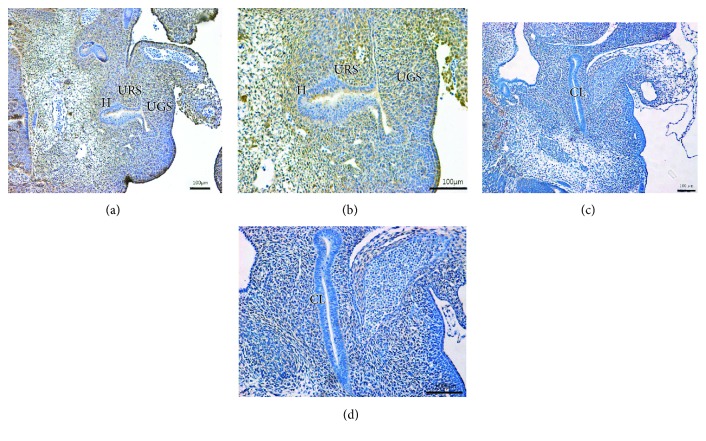
(a, b) On E13, normal embryos had typical cloacal morphology, and URS divided the cloaca into UGS and hindgut. Abundant Fgf9 immunopositive cells were present in the cloacal mesenchyme. (c, d) In ARM embryos, Fgf9-positive cells were rarely present in the mesenchyme. URS: urorectal septum; UGS: urogenital sinus; H: hindgut; CL: cloaca. Original magnification: ×100 (a, c); ×200 (b, d).

**Figure 2:**
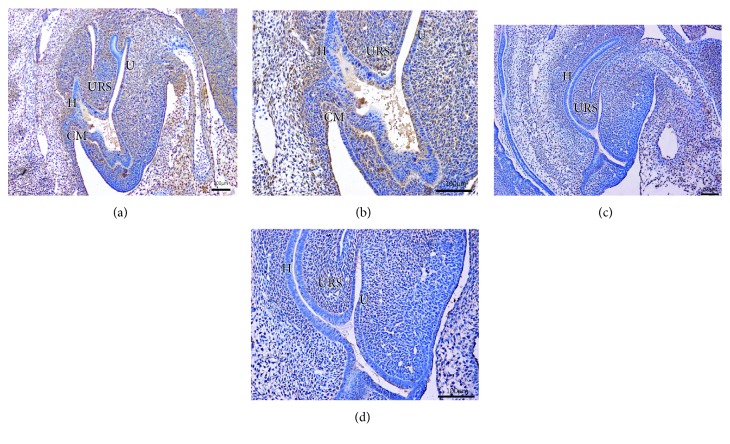
(a, b) On E14, URS divided the cloaca into the urogenital sinus and hindgut clearly. Fgf9-positive cells were mainly present in the mesenchyme, and a few positive cells were seen in the intestinal epithelium and CM. (c, d) In the ARM embryos, the distance from the hindgut to CM was relatively long. Staining of Fgf9-positive cells in the mesenchyme was faint. URS: urorectal septum; H: hindgut; U: urethra; CM: cloacal membrane. Original magnification: ×100 (a, c); ×200 (b, d).

**Figure 3:**
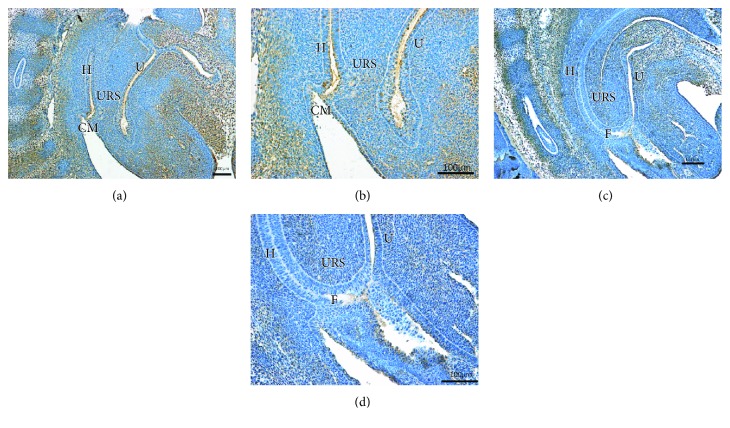
(a, b) On E15, the epithelium of the URS in normal embryos had fused with CM, leading to the separation of the hindgut and urethra. Fgf9 immunolabeled cells were expressed mainly in the rectal epithelium, CM, and the fusion site of URS and CM. (c, d) In the ARM embryos, there was a fistula between the hindgut and urethra. Fgf9-positive cells were scattered within the mesenchyme of URS and were not present in the epithelium. URS: urorectal septum; H: hindgut; U: urethra; CM: cloacal membrane; F: fistula). Original magnification: ×100 (a, c); ×200 (b, d).

**Figure 4:**
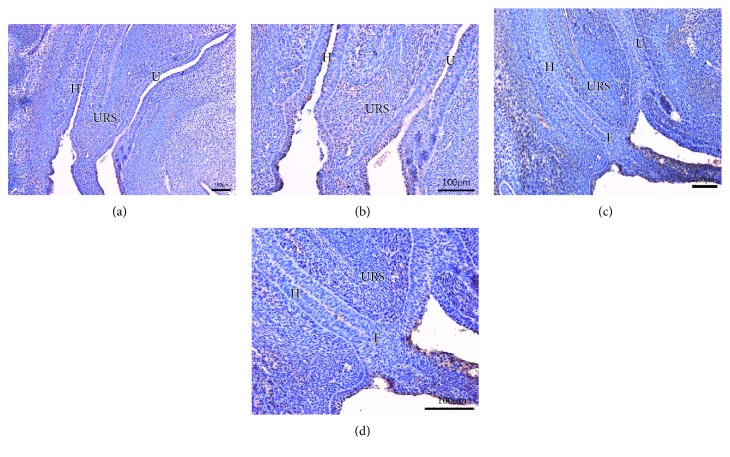
(a, b) On E16, the cloacal membrane ruptured and the rectum communicated with the outside world in normal embryos. Fgf9-positive cells were seen at the intestinal epithelium and the fusion area, but the intensity of immune reaction was obviously weakened. (c, d) In the ARM embryos, the URS had not fused with the cloacal membrane; there was only sporadic Fgf9 cell distribution in the mesenchyme of URS and rectum. URS: urorectal septum; H: hindgut; U: urethra; F: fistula. Original magnification: ×100 (a, c); ×200 (b, d).

On E13 in the normal group, the cloaca had formed at the end of the tail, and the urorectal septum (URS) divided the cloaca into the urogenital sinus (UGS) and the primitive rectum (hindgut). Abundant Fgf9-immunopositive cells were present in the cloacal mesenchyme, and some positive cells were expressed in the epithelium of the hindgut (Figures 1(a) and 1(b)). On E14, the URS descended and divided the cloaca into the UGS and hindgut clearly. Fgf9-positive cells were primarily expressed in mesenchyme, including the URS and hindgut. A few positive cells were seen in the intestinal epithelium and around the opening of the anus (Figures 2(a) and 2(b)). On E15, the epithelium of the URS was fused with the cloacal membrane (CM), leading to the separation of the rectum and the urethra. The CM had not been broken. Many Fgf9-positive cells were seen at the site of fusion of the URS and CM. Strongly positive cells were present in the epithelium of the colon and rectum, especially in the terminal rectum and anal membrane (Figures 3(a) and 3(b)). On E16, the rectum was completely separated from the urethra, and the anal membrane ruptured. The rectum communicated with the outside world. Fgf9-positive cells were seen in the epithelium of the colon and rectum and at the site of fusion of the URS and CM, but the positive cells were obviously decreased (Figures 4(a) and 4(b)).

On E13 in the ARM group, the cloaca did not have the usual structure, and Fgf9-positive cells were rarely present in the mesenchyme and epithelium (Figures 1(c) and 1(d)). On E14, the distance from the hindgut to the CM was relatively long, and the mesenchyme included weakly positive Fgf9 cells (Figures 2(c) and 2(d)). On E15, a fistula was evident between the rectum and urethra, and the URS had not fused with the CM. Fgf9-positive cells were scattered within the mesenchyme of URS, and none were present in the rectal epithelium (Figures 3(c) and 3(d)). On E16, the URS had not fused with the CM; there were only sporadic Fgf9 cells in the mesenchyme of URS and rectum (Figures 4(c) and 4(d)).

### 3.3. Western Blot Assay

**Figure 5:**
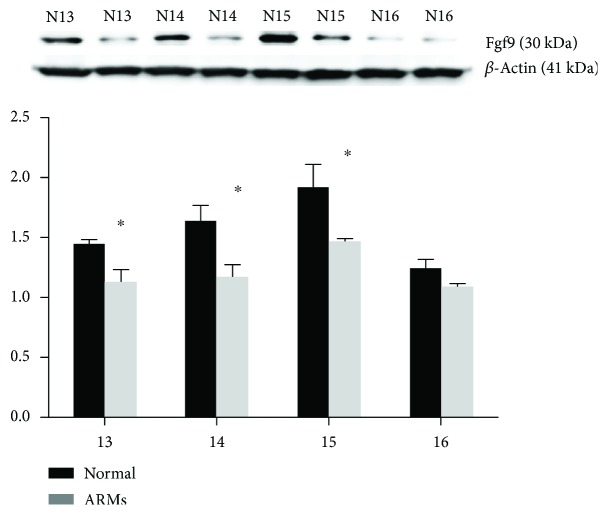
Western blots showed that Fgf9 expression increased in normal embryos from E13 to E15 and that expression in the ARM embryos was weak. On E16, the expressions of the two groups were decreased. Values were presented as means ± SD. ^∗^Significant difference from corresponding controls.

Fgf9 protein expression during the development of the hindgut and rectum was assayed by Western blotting. Fgf9 was detected as a 30 kDa band on blots of protein extracted from both normal and ARM tissues. Each band was normalized against a corresponding *β*-actin band. Fgf9 expression increased from E13 to E15 in the normal rat embryos but was comparatively low in the ARM embryos. The differences in Fgf9 protein expression between the two groups were significant from E13 to E15 (Figure 5). On E13, relative expression was 1.45 ± 0.03 in normal embryos and 1.13 ± 0.09 in ARM embryos. The corresponding values on E14 were 1.65 ± 0.12 and 1.18 ± 0.10 and were 1.92 ± 0.18 and 1.48 ± 0.01 on E15. On E16, the expression of Fgf9 began to decrease in the normal and ARM groups, and there was no statistical difference between the two groups. The relative expression was 1.25 ± 0.07 in normal embryos and 1.11 ± 0.02 in ARM embryos.

### 3.4. qRT-PCR Assay

**Figure 6:**
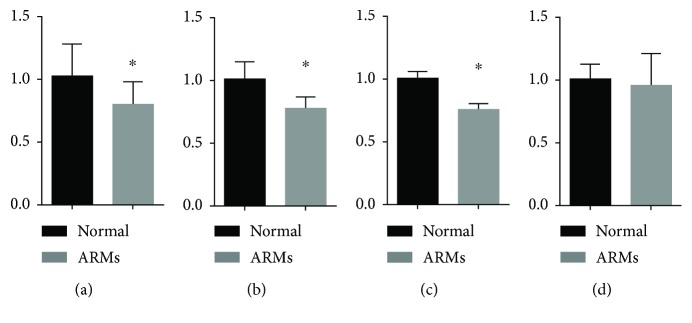
qRT-PCR assayed Fgf9 mRNA expression in the hindgut and rectum of normal and ARM embryos. (a–c) Fgf9 mRNA expression was significantly higher in normal embryos than that of ARM embryos from E13 to E15. (d) On E16, there was no difference between the two groups. Values were presented as means ± SD. ^∗^Significant difference from corresponding controls.

Fgf9 mRNA expression was assayed in the hindgut and rectum of normal and ARM rats. Fgf9 mRNA expression was significantly higher in normal embryos than ARM embryos from E13 to E15, as calculated by 2^−*ΔΔ*CT^ (E13: 1.02 ± 0.25 vs. 0.79 ± 0.18; E14: 1.0 ± 0.14 vs. 0.77 ± 0.09; E15: 1.0 ± 0.06 vs. 0.75 ± 0.04; *P* < 0.05) (Figures 6(a)–6(c)). On E16, there was no difference between the two groups (E16: 1.0 ± 0.12 vs. 0.95 ± 0.25; *P* > 0.05) (Figure 6(d)).

## 4. Discussion

ARM is a pathological process that is caused by complex factors. Although there are heated debates between scholars who hold different views, it is generally believed that ARM is associated with dysplasia of cloaca and the failure of fusion of URS with CM [14, 16]. The fusion of URS with CM is essential for the separation of the rectum from the urethra on E15 in rat embryos. On E16, the anal membrane ruptures, and the rectum communicates with the outside. In ETU-induced ARM rat embryos, URS never fused with CM and the dorsal CM was maldeveloped.

In this study, we studied the spatial and temporal expression patterns of Fgf9 during the normal anorectal development by immunohistochemical staining, Western blot analysis, and real-time RT-PCR. Fgf9-positive cells were mainly expressed in the mesenchyme of URS and hindgut on E13 and E14 by immunohistochemistry. On E15, immunoreactive cells were mainly found in the epithelium of the rectum, the anal membrane, and the site of fusion of the URS and CM. On E16, the Fgf9-positive cells were markedly weakened. Western blotting and qRT-PCR revealed that the expression of Fgf9 protein and mRNA increased from E13 to E15, but Fgf9 expression decreased after the anal membrane ruptured. Such a high specific Fgf9 spatiotemporal expression revealed that Fgf9 may play a key role during the anorectal development.

There was an imbalance in the spatiotemporal expression of Fgf9 during the development of ARM. On E13 and E14, Fgf9-positive cells were rarely present in the cloacal mesenchyme. On E15, Fgf9-positive cells were scattered within the URS, and none were present in the rectal epithelium. On E16, the anal membrane had not ruptured; there were only sporadic Fgf9 cells in the mesenchyme of URS and rectum. Western blotting and qRT-PCR revealed that the expression of Fgf9 protein and mRNA significantly reduce in the hindgut and rectum of ARM embryos than that in normal embryos from E13 to E15. These results suggest that Fgf9 participated in the formation of the anorectum and that abnormal expression of Fgf9 may involve in the morphogenesis of ARM.

Fgf9 is an important member of the Fgf signaling pathway which mediates reciprocal mesenchymal-epithelial cell interactions during embryogenesis. Fgf9 is first reported in a human glioma cell line and is a secreted polypeptide that is active in lung and bone development and steroidogenesis in postnatal Leydig cells [17–20]. It is highly conserved, with >93% sequence identity in Xenopus, mice, rats, and humans [21], indicating that Fgf9 is evolutionarily important and may have similar functions across species.

Animal experiments have shown that Fgf10 is critical for normal anorectal development, and Fgf10 invalidation results in genetically reproducible ARM [10]. But the deformity is of a single type; there is no rectal epithelium and no fistula between the rectum and urinary tracts. It is presumed that isolated ARM in the absence of other anomalies may occur not as a result of deletion of the Fgf10 gene itself but rather as a mutation of the regulatory elements controlling Fgf10 expression in the rectum. Fgf9 induces proliferation of the mesenchyme and upregulates mesenchymal Fgf10 expression during the development of the cecum and lung [12, 22–24]. Fgf9-Fgf10 reciprocal epithelial-mesenchymal cell interactions are essential to embryonic development of the cecum and lung. And the expression of Fgf9 influences the function of Bmp4 and Wnt pathway which are crucial to anorectal development [23, 24]. We hypothesized that Fgf9 might affect the development of the anorectum as a transcription factor by regulating the expression of Fgf10.

We observed a significant difference in the expression of Fgf9 between normal and ARM embryos during the development of the anorectum. The results showed that the anorectal morphology may depend on the induction of Fgf9 signal, and the downregulation of Fgf9 may be one of the reasons for ARM.

## Figures and Tables

**Table 1:** Distribution of embryos from E13-E16 in the normal group.

	E13	E14	E15	E16
Total number	63	57	53	50
IHC	22	20	17	16
WB	20	19	18	17
qRT-PCR	21	18	18	17

E: embryonic day; IHC: immunohistochemical staining; WB: Western blot; qRT-PCR: real-time quantitative polymerase chain reaction.

**Table 2:** Distribution of embryos from E13-E16 in the ARM group.

	E13	E14	E15	E16
Total number	59	56	51	48
IHC	20	19	17	15
WB	19	20	17	15
qRT-PCR	20	17	17	18

E: embryonic day; ARM: anorectal malformations; IHC: immunohistochemical staining; WB: Western blot; qRT-PCR: real-time quantitative polymerase chain reaction.

## Data Availability

The data used to support the findings of this study are included within the article.
